# Emotional, Social, and Existential Loneliness Before and During the COVID-19 Pandemic: Prevalence and Risk Factors Among Dutch Older Adults

**DOI:** 10.1093/geronb/gbab101

**Published:** 2021-06-07

**Authors:** Theo G van Tilburg

**Affiliations:** Department of Sociology, Vrije Universiteit Amsterdam, The Netherlands

**Keywords:** Disaster, Longitudinal methods, Structural equation models

## Abstract

**Objectives:**

The coronavirus disease 2019 pandemic, with its accompanying isolation measures, has led to increasing loneliness among older adults. In this study, we examine whether the increased level of loneliness observed in the Netherlands persisted into the fall of 2020, whether there were differences in emotional, social, and existential loneliness, and whether the presence of well-known risk factors for loneliness also led to further increases in loneliness during the pandemic.

**Methods:**

Data were obtained from the Longitudinal Aging Study Amsterdam, with observations of 404 community-dwelling older adults aged 74–96 years from 2019 and fall 2020.

**Results:**

Loneliness increased between 2019 and 2020, and the increase was particularly high for emotional loneliness (partial η ^2^ = 0.19). Having a partner and a high mastery and good physical functioning before the pandemic provided some protection against an increase in loneliness.

**Discussion:**

Loneliness increased for almost all older people. Targeted policies can reduce the negative impact of vulnerabilities. Efforts to combat loneliness during the pandemic should focus not only on groups traditionally considered vulnerable, such as socially isolated people, but also on older adults with a partner and who have daily contact with others.

The coronavirus disease 2019 (COVID-19) pandemic, with its accompanying social and physical isolation measures, has led to increasing loneliness among older adults aged 65 to older than 100 according to longitudinal studies with measurements from before and during the first phase of the pandemic in 2020 in the United States (Krendl & Perry, 2021; Luchetti et al., 2020), Switzerland (Macdonald & Hülür, 2021), and Norway (Hansen et al., 2021). In the Netherlands, an increase in emotional loneliness was particularly noted (Knapen et al., 2020; van Tilburg et al., 2020). In the early days after the pandemic outbreak, no increase was observed among adults aged 65–71 in Sweden, which had a low infection rate at the time and had imposed less severe measures (Kivi et al., 2021), and a loneliness decline was observed in a sample with an average age of 55 from Spain (Bartrés-Faz et al., 2021). In this study, we examine whether the increased level of loneliness observed in the Netherlands persisted into the fall of 2020; whether there were differences in emotional, social, and existential loneliness; and whether the presence of well-known risk factors for loneliness also led to further increases in loneliness during the pandemic.

The cognitive approach to loneliness involves weighing the quantity and quality of social relationships against certain relationship standards (Perlman & Peplau, 1981): Loneliness is “the unpleasant experience that occurs when a person’s network of social relations is deficient in some important way” (p. 31). Emotional loneliness originates from situations in which the relationships’ quality or intimacy one desires has not been realized. Social loneliness originates from the absence of being embedded in a broader group of contacts. Existential loneliness is a sense of meaninglessness in life (van Tilburg, 2020).

In the pandemic, when it becomes impossible to be in close contact with loved ones, emotional loneliness arises (van Tilburg et al., 2020). Furthermore, people realized that meeting with family and friends was practically impossible. Therefore, social loneliness did not increase extensively for everyone and even decreased for some (Bartrés-Faz et al., 2021). The increase in social loneliness may also have been countered by the fact that people experienced solidarity: fate affected everyone and it was necessary to fight the effects of the pandemic together (Luchetti et al., 2020). Existentially lonely people realize that they are fundamentally separated from social life, and the pandemic may have increased their loneliness. Hypothesis 1 is that loneliness increased with the development of the pandemic, with the strongest increase being the increase in emotional loneliness.

The antecedents of loneliness are a lack of social integration (e.g., a small personal network, infrequent contact with others, and a lack of participation in social organizations) and of resources, that is, factors that shape the characteristics of individuals’ living conditions and consequently affect their level of social integration (de Jong Gierveld et al., 2018; Tesch-Roemer & Huxhold, 2019). A better health and a higher mastery, self-esteem, educational level, and income decrease the likelihood of loneliness. Loneliness is more often observed among the oldest. Gender differences are often absent when social integration and resources are taken into account. In a longitudinal study, Aartsen and Jylhä (2011) showed that aging-related relationship losses and resource losses were related to enhanced feelings of loneliness rather than to respondent characteristics at baseline. The pandemic and social distancing measures are a historically unique situation in which people have difficulty accessing social networks and resources, leading to increased loneliness. It is also possible that social integration and resources from before the pandemic outbreak are sustainable and still provide protection against the occurrence or increase of loneliness during the pandemic. Hypothesis 2 is that older adults who were socially integrated and had access to resources were less prone to increased loneliness during the pandemic.

We analyze data from older adults in the Netherlands. The earliest severe acute respiratory syndrome coronavirus 2 infections were in February 2020. The rapid increase in COVID-19 cases forced the government to take firm action in mid-March. Many public activities were restricted or canceled, restaurants and cafes were closed, and many stores closed or offered limited access. Due to a lack of hospital capacity, most regular care was halted. Personal meetings at home were limited in number and frequency. Outdoor activities could continue. After infection, an in-home quarantine was mandatory. From mid-May to early July, many measures were relaxed. In September, the second wave of infections and diseases started. There was a partial lockdown in October; then, the measures became more severe, but individuals could still be outside. In 2020, excess mortality was 10% compared to 2019.

## Method

### Respondents

Data were obtained from the Longitudinal Aging Study Amsterdam (Huisman et al., 2011). Samples of men and women born between 1908 and 1957 were taken from the population registers of three cities and six surrounding small municipalities in 1992, with additions in 2002 and 2012. Home or telephone interviews were completed with 1,591 men and women in 2019. Respondents born in 1945 or earlier (*N* = 404) were selected (Supplementary Figure S1) and interviewed in the fall of 2020. Home interviews were planned, but due to pandemic-related government measures, other modes were also used. In 2020, their ages ranged from 74 to 96 (*M* = 81.2), and 53% were women. Most respondents were of Dutch origin. The majority (57%) lived with their partner with no others in the household, nine lived with another person and 41% lived alone.

### Measures

Supplementary Table S1 presents the loneliness questions. Three direct loneliness questions were asked (van Tilburg, 2020). Six and five items were used to measure emotional and social loneliness, respectively (de Jong Gierveld & Kamphuis, 1985). Existential loneliness was measured by seven items (Mayers et al., 2002; van Tilburg, 2020).

Partner status was assessed, and network members with whom there was regular contact and who were important to the respondent were identified by name (van Tilburg, 1998). We derived the personal network size (not counting the partner) and whether the respondent was in daily contact with someone in the network (to avoid collinearity, respondents who lived with a partner were assigned a zero). For social participation, we measured the total frequency with which meetings of 12 societal organizations were visited (e.g., patients association, choir) on a scale from “never” to “a few times a week or more.” The frequency of church attendance was assessed on a scale from “never” to “once a week or more.” Mastery was measured with five items (Pearlin & Schooler, 1978); Cronbach’s alpha = 0.76. Self-esteem was measured with four items (Rosenberg, 2015); alpha = 0.70. We counted the number of chronic diseases in seven major categories. Physical functioning was measured by six items (Katz et al., 1963); alpha = 0.81. Level of education varies from “not completed” to “university” education and was converted to years of education. Monthly net income was measured in 24 categories and recoded into four categories. The level of urbanity was derived from Statistics Netherlands. Ranges and means are displayed in Table 1.

**Table 1.** Regression of Loneliness at T1 and T0 (standardized estimates)

	Range	*M*	Emotional		Social		Existential	
			T1	T0	T1	T0	T1	T0
Emotional T0			0.52***					
Social T0					0.47***			
Existential T0							0.45***	
Living with partner T0	0–1	0.57	−0.23***	−0.34***	−0.15**	−0.23***	−0.19***	−0.26***
Network size T0	0–60	15.3	0.04	−0.06	−0.07	−0.19***	−0.02	−0.14**
Daily contacted network member T0	0–1	0.22	−0.02	0.00	0.00	−0.12*	−0.03	−0.08
Social participation frequency T0	0–6	3.0	−0.07	−0.06	−0.08*	−0.06	−0.05	−0.10*
Church attendance frequency T0	1–6	2.6	0.09*	0.01	−0.01	−0.01	0.02	−0.07
Mastery T0	5–25	17.6	−0.15***	−0.13**	−0.16***	−0.11*	−0.14**	−0.07
Self-esteem T0	4–20	15.5	−0.01	−0.19***	−0.06	−0.20***	−0.05	−0.23***
Number of chronic diseases T0	0–7	1.6	0.01	0.03	0.03	−0.02	0.03	0.04
Physical functioning T0	6–30	26.8	−0.11**	−0.07	−0.10*	−0.07	−0.10*	−0.09
Educational level (years)	5–18	10.8	0.01	−0.02	0.05	−0.01	0.02	0.00
Income T0	1–4	2.8	−0.03	0.03	0.01	0.01	−0.05	0.04
Level of urbanity T0	1–5	3.2	−0.01	0.01	−0.08	0.02	−0.01	0.02
Age T0	72–94	79.5	−0.05	0.04	−0.09*	0.06	−0.04	0.05
Female	0–1	0.53	−0.07	0.00	−0.04	−0.04	−0.02	0.03
Interval T0–T1 (years)	1.1–2.2	1.7	−0.03		−0.05		−0.06	
Variation interview date T1 (days)	−46 to 71	0.0	0.02		−0.02		0.01	
Oral interview T1	0–1	0.67	−0.01		−0.15***		−0.08*	
*R* ^2^			0.53	0.30	0.45	0.23	0.45	0.36

*Notes: N* = 404. Root mean square error of approximation = 0.061, comparative fit index = 0.991. At T0, emotional loneliness correlates 0.67 and 0.79 with social and existential loneliness, respectively; social correlates 0.79 with existential. At T1, emotional loneliness correlates 0.63 and 0.76 with social and existential loneliness, respectively; social correlates 0.75 with existential.

*p < .05, **p < .01, ***p < .001.

### Procedure

We applied confirmatory factor analysis in Mplus (Muthén & Muthen, 2017) to assess the existence of three correlated loneliness factors on the pooled data from both observations. The first factor combined direct and emotional loneliness items because of the high correlations (van Tilburg, 2020), the other factors were social and existential loneliness. Hypothesis 1 was tested by assessing longitudinal differences in item and factor scores, controlled for three design characteristics: length of the interval T0–T1, variation in T1 interview date, and the T1 mode of interview. Structural equation modeling in Mplus was used to test Hypothesis 2. Twelve variables for social integration and resources were the explanatory factors for differences in three loneliness dimensions at T0 and T1. The regression of T1 loneliness is controlled for T0 loneliness, age at T0, gender, and the three design characteristics. We imputed few missing values and created 10 data sets, presenting the pooled estimates. Model fit was evaluated by the root mean square error of approximation and the Tucker–Lewis Index (Hu & Bentler, 1999).

## Results

The three-factor model for loneliness fitted the data well (Supplementary Table S1). Factor loadings for emotional loneliness items were high, and loadings for the existential loneliness items were relatively low.

Supporting Hypothesis 1, in the fall of 2020, the respondents were lonelier than they were 1.7 years earlier. A large increase was observed for emotional loneliness (partial η ^2^ = 0.19; Supplementary Table S2). The increase was medium for social (partial η ^2^ = 0.13) and existential loneliness (partial η ^2^ = 0.11). The increase in agreement with the 21 loneliness items averaged seven percentage points (Supplementary Tables S3 and S4).

For example, for emotional loneliness agreement with the item, “I miss having people around me” increased by 20 percentage points, and missing the pleasure of the company of others increased by 23 percentage points (Supplementary Table S4). Increases in prevalence and effect sizes for these two items were also high in the subsamples of those who lived with a partner, those who did not live with a partner but had daily contact with a network member, and those without a partner and without daily contact. For one emotional, two social, and two existential loneliness items, the change over time differed by the mode of the interview.

The regression model of loneliness had a good fit (Table 1; correlations are given in Supplementary Table S5). For the test of Hypothesis 2, the T1 loneliness score regression was relevant; the cross-sectional associations between T0 loneliness and other T0 variables are not discussed. From T0 to T1, the parameters of the T0 loneliness scores indicate that many of those relatively high in loneliness before the pandemic were among the loneliest respondents during the pandemic. Despite this persistence, social integration was important. Living with a partner protected against increasing emotional, social, and existential loneliness. Social participation before the outbreak of the pandemic protected against an increase in social loneliness. However, frequent church attendance before the pandemic increased the likelihood of emotional loneliness during the pandemic. Of the resources assessed, only a high mastery and good physical functioning protected against an increase of loneliness, in all three forms.

## Discussion

During the second wave of the pandemic, loneliness among Dutch older adults was still high. The increase in agreement with loneliness items averaged 7 percentage points, and the proportion of respondents who classified themselves as lonely increased from 23% to 28% between early 2019 and fall 2020.

Most previous longitudinal studies have not distinguished between different dimensions of loneliness. The increase we observed was particularly high for emotional loneliness, which stems from the absence of a close emotional bond such as with a partner (de Jong Gierveld et al., 2018). This origin is reflected in the difference by partner status. However, we found that the increase in emotional loneliness was high for everyone regardless of partner status and having daily network contact. A protective factor was higher mastery, a factor that along with high self-esteem also protected before the pandemic. Better physical functioning protected; the effect was somewhat stronger than before the pandemic. Those resources helped to shape social life in a time of all kinds of restrictions. Those with a higher frequency of church attendance before the pandemic were more emotionally lonely during the pandemic. It may be that they missed the “live” church services and that the online variants did not provide the same level of connectedness.

Social loneliness stems from the absence of a broader group of contacts or an engaging social network. We saw an increase in social loneliness, smaller than that in emotional loneliness. Some suffer from the lockdown, while for others the effects have been mitigated by continued or increasing contact with others. Macdonald and Hülür (2021) found that only dissatisfaction with communication during the pandemic was related to a relatively small increase in loneliness. Whether contact without in-person meetings, for example, through the Internet, phone calls, and writing letters, or adjusting to restrictions and lowering standards for the possibility of having frequent in-person contact is related to pandemic-related changes in loneliness has not been studied longitudinally. We find that also for social loneliness, living with a partner, higher mastery, and better physical functioning is protective. Social participation before the pandemic is also protective: It apparently provides structurally embedded connectedness and social contact even when virtually all social activities have been shut down due to contagion risk. However, a large social network and having daily contact, protective in the cross-sectional analysis of the prepandemic situation, did not help against the increase in social loneliness during the pandemic. One reason may be that these forms of social participation were under pressure from the social distancing measures because they are less structurally embedded. Alternatively, those close social contacts were easier to limit in the expectation that they would weather the pandemic. The relatively young older respondents were more vulnerable to social loneliness, perhaps because they wanted to achieve more and had higher relationship standards.

For protection against existential loneliness, again living with a partner, higher mastery, and better physical functioning are important. On average, there was almost no change in agreement with the items and we found a medium effect size for the increase in existential loneliness during the pandemic. One might have expected more pressure on finding meaning in life. According to Stickley and Koyanagi (2016), the answer to loneliness has always been to be at peace with oneself. In times of crisis and suffering, individuals should be understanding toward themselves. This self-compassion (Neff, 2011) can help maintain well-being in times of increased stress and societal isolation (Schnepper et al., 2020). This is consistent with the cognitive approach to loneliness, which states that in response to loneliness, one could lower expectations and be satisfied with the social contact that can be maintained.

Loneliness was measured with three dimensions, of which emotional loneliness is the core which is related to how respondents interpret items containing the word “lonely” or “loneliness.” Existential loneliness is not measured homogeneously and has a somewhat diffuse content. Emotional, and to a lesser extent social, loneliness increased because of the pandemic. The three forms correlate strongly, and risk factors for loneliness during the pandemic are predominantly the same for emotional, social, and existential loneliness.

We selected common risk factors for loneliness and found that some led to further increases in loneliness during the pandemic. In contrast to the findings of Bu et al. (2020), we did not observe that older people in cities had a higher risk of being lonely, we did not find that socioeconomic differences changed, and in our study, some risk factors for loneliness were different in the cross-sectional analysis of data from before the pandemic and in the analysis of changes during the pandemic. Congruent with the literature, we observed that men and women had similar levels of loneliness (Maes et al., 2019). Lower loneliness was observed in oral interviews than in written and digital questionnaires (Tourangeau & Smith, 1996). We found no differences in loneliness related to the period of data collection.

This study has some limitations. Because our results are also in line with those of Dutch research on loneliness during the first wave, it seems that average loneliness did not fluctuate much after the pandemic outbreak, but we did not investigate this. The observation in fall 2020 that we used was a short survey, and we were therefore not able to study changes in risk factors and coping behaviors.

In conclusion, during this pandemic, loneliness, and in particular, emotional loneliness, increased among community-dwelling older people. Protective factors that were important before the pandemic (having a partner, a high mastery, and good physical functioning) were also so during the pandemic. Vulnerabilities in these areas are difficult to change, but targeted policies can reduce their negative impact. This is always important, but also specifically during this pandemic. It is also interesting to develop policies that make social integration that is effective prepandemic also effective during the pandemic. The isolation measures affected not only older people living alone who were less socially integrated but also older people who lived together with a partner or had daily contact with a network member. Efforts to combat loneliness should not, therefore, focus only on groups traditionally seen as vulnerable.

## Supplementary Material

gbab101_suppl_Supplementary_Material
