# Supplementary material for: Emotional, Social, and Existential Loneliness Before and During the COVID-19 Pandemic: Prevalence and Risk Factors Among Dutch Older Adults
Source: J Gerontol B Psychol Sci Soc Sci. 2021 Jun 7;77(7):e179–84. doi: 10.1093/geronb/gbab101 (PMC8394819; doi:10.1093/geronb/gbab101)
Supplement: gbab101_suppl_Supplementary_Material [file gbab101_suppl_supplementary_material.pdf]

Emotional, social, and existential loneliness before and during the COVID-19 pandemic: Prevalence and risk factors among Dutch older adults

Supplementary Material

Theo G. van Tilburg, PhD

Dept. of Sociology, Vrije Universiteit Amsterdam

Theo.van.Tilburg@vu.nl

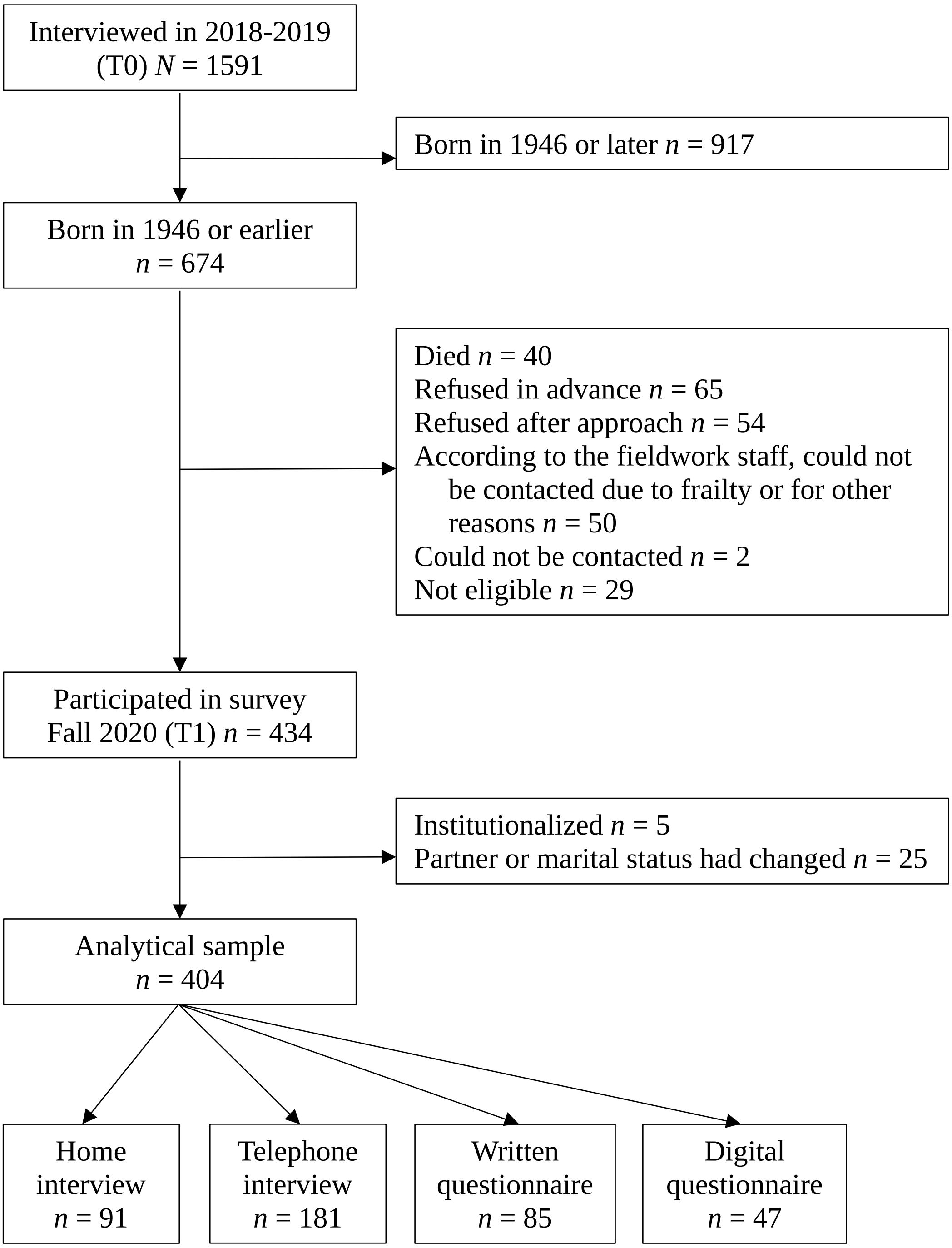

Figure S1. Flow chart summarizing the selection of respondents
Notes. Interviews at T0 were between October 11, 2018, and October 21, 2019; the mean interview date was February 15. Data collection at T1 was between September 23, 2020, and January 18, 2021; the mean response date was November 8.

Table S1. Factor loadings for loneliness items

Emotional loneliness	
If we were to classify people into not … very strongly lonely. what would you attribute yourself to?	0.93
I sometimes feel lonely	0.94
During the past week I felt lonely	0.90
I miss having a really close friend	0.71
I experience a general sense of emptiness	0.89
I miss the pleasure of the company of others	0.87
I find my circle of friends and acquaintances too limited	0.79
I miss having people around me	0.88
I often feel rejected	0.84
Social loneliness	
There is always someone I can talk to about my day-to-day problems †	0.76
There are plenty of people I can lean on when I have problems †	0.83
There are many people I can trust completely †	0.73
There are enough people I feel close to †	0.85
I can call on my friends whenever I need them †	0.81
Existential loneliness	
I am happy with the way I have lived my life †	0.51
There is a purpose to my life †	0.32
I feel helpless	0.87
I feel at the mercy of the world	0.44
I feel dead	0.90
The universe is full of meaning †	0.44
I feel that there is little point to life	0.65

Notes. N = 404. Scores are reversed for positively phrased items marked with †. Items are dichotomous. Fit for the three-factor model: RMSEA = .046, CFI = .972. Emotional factor scores correlate 0.64 and 0.71 with social and existential factor scores, respectively; social correlates 0.69 with existential.

Table S2. General linear modelling (repeated measures) of loneliness factor scores

	Within and between respondents effects				Estimated marginal means											
					Written or digital questionnaire				Home or telephone interview				All			
	Observation		Mode	Observation * Mode	T0		T1		T0		T1		T0		T1	
	F	partial η^2	*F*	*F*	M	SE	M	SE	M	SE	M	SE	M	SE	M	SE
Emotional	95.4 ***	0.19	6.9 **	0.7	-0.14	0.06	0.15	0.06	0.07	0.04	0.31	0.04	-0.03	0.03	0.23	0.04
Social	59.5 ***	0.13	0.0	17.3 ***	-0.05	0.05	0.25	0.05	0.06	0.03	0.15	0.03	0.00	0.03	0.20	0.03
Existential	47.6 ***	0.11	1.8	7.1 **	-0.04	0.03	0.11	0.03	0.05	0.02	0.12	0.02	0.01	0.02	0.11	0.02

Notes. $N = 404$. Written or digital questionnaire, $n = 132$; home or telephone interview, $n = 272$. Covariates are length of the interval T0-T1 and variation in T1 interview date (effects not shown). Based on Cohen's (1988) *f*, $\eta^2 = 0.01$ indicates a small effect; $\eta^2 = 0.06$ indicates a medium effect; $\eta^2 = 0.14$ indicates a large effect.

* $p < .05$; ** $p < .01$; *** $p < .001$.

Table S3. Generalized estimating equations regression of item scores: model effects and parameter estimates

	Within and between respondents model effects			Parameter estimates		
	Observation χ^2	Mode χ^2	Observation * Mode χ^2	Observation B	Mode B	Observation * Mode B
Emotional loneliness						
If we were to classify people into not … very strongly lonely, what would you attribute yourself to?	3.8	3.0	0.1	0.185	0.339	0.087
I sometimes feel lonely	13.6 ***	7.1 **	0.3	0.342	0.522 *	0.119
During the past week I felt lonely	3.4	10.7 **	1.6	0.501	0.995 **	-0.406
I miss having a really close friend	2.1	0.2	3.7	0.442 *	0.359	-0.504
I experience a general sense of emptiness	17.4 ***	8.1 **	1.3	0.830 **	0.895 **	-0.361
I miss the pleasure of the company of others	69.8 ***	4.1 *	0.4	1.037 ***	0.351	0.162
Not living with partner, no daily contact with others	11.0 ***	0.0	1.1	0.566	-0.183	0.523
Not living with partner, daily contact with others	8.1 **	0.1	0.9	0.498	-0.092	0.512
Living with partner	42.2 ***	3.2	0.7	2.306 ***	0.984	-0.547
I find my circle of friends and acquaintances too limited	36.6 ***	0.5	0.0	0.731 ***	0.140	0.027
I miss having people around me	54.6 ***	7.8 **	0.3	0.945 ***	0.538	0.143
Not living with partner, no daily contact with others	20.7 ***	0.1	0.2	0.992 *	0.012	0.242
Not living with partner, daily contact with others	10.4 **	0.6	0.0	1.057 *	0.399	-0.134
Living with partner	25.7 ***	4.7 *	0.2	1.153 **	0.655	0.199
I often feel rejected	15.4 ***	0.0	4.1 *	1.517 ***	0.491	-1.034 *
Social loneliness						
There is always someone I can talk to about my day-to-day problems †	15.8 ***	0.9	10.6 **	1.396 ***	0.881 *	-1.258 **
There are plenty of people I can lean on when I have problems †	7.0 **	1.3	3.7	0.834 **	0.069	-0.703
There are many people I can trust completely †	9.5 **	1.1	2.1	0.588 **	-0.011	-0.378
There are enough people I feel close to †	7.7 **	0.0	3.4	0.687 **	0.298	-0.548
I can call on my friends whenever I need them †	23.3 ***	0.6	10.2 **	1.272 ***	0.325	-1.013 **
Existential loneliness						
I am happy with the way I have lived my life †	0.8	0.1	0.0	0.191	0.104	-0.070
There is a purpose to my life †	0.4	3.2	3.3	0.268	-0.156	-0.396
I feel helpless	2.5	0.4	0.8	0.165	0.029	0.441
I feel at the mercy of the world	1.3	2.8	8.5 **	0.227	0.702 **	-0.735 **
I feel dead	1.1	0.3		0.522	0.334	
The universe is full of meaning †	6.3 *	0.7	5.0 *	0.631 **	0.129	-0.594 *
I feel that there is little point to life	0.0	0.0	0.0	0.000	0.022	0.000

Notes. *N* = 404. Not living with partner, no daily contact with others, *n* = 86; not living with partner, daily contact with others, *n* = 87; living with partner, *n* = 231. Scores are reversed for positively phrased items marked with †. Items are dichotomous. Covariates are length of the interval T0-T1 and variation in T1 interview date (model effects and parameter estimates not shown). For the item "I feel dead," the convergence criteria for the interaction model were not met.
* $p < .05$; ** $p < .01$; *** $p < .001$.

Table S4. Generalized estimating equations regression of item scores: estimated marginal means

	Written or digital questionnaire				Home or telephone interview				All				
	T0		T1		T0		T1		T0		T1		*d*
	M	SE	M	SE	M	SE	M	SE	M	SE	M	SE	
Emotional loneliness													
If we were to classify people into not … very strongly lonely, what would you attribute yourself to?	0.20	0.04	0.24	0.04	0.26	0.03	0.32	0.03	0.23	0.02	0.28	0.02	0.10
I sometimes feel lonely	0.20	0.03	0.27	0.04	0.30	0.03	0.41	0.03	0.25	0.02	0.33	0.03	0.19
During the past week I felt lonely	0.11	0.03	0.18	0.03	0.26	0.03	0.28	0.03	0.17	0.02	0.22	0.02	0.28
I miss having a really close friend	0.19	0.03	0.26	0.04	0.25	0.03	0.24	0.03	0.21	0.02	0.25	0.02	0.24
I experience a general sense of emptiness	0.09	0.02	0.19	0.03	0.20	0.03	0.29	0.03	0.14	0.02	0.24	0.02	0.46
I miss the pleasure of the company of others	0.16	0.03	0.35	0.04	0.22	0.03	0.48	0.03	0.19	0.02	0.41	0.03	0.57
Not living with partner, no daily contact with others	0.42	0.11	0.56	0.10	0.38	0.06	0.64	0.06	0.40	0.06	0.60	0.06	0.31
Not living with partner, daily contact with others	0.37	0.10	0.50	0.10	0.35	0.06	0.60	0.07	0.36	0.06	0.55	0.06	0.28
Living with partner	0.03	0.02	0.26	0.05	0.08	0.02	0.35	0.04	0.05	0.02	0.30	0.03	1.27
I find my circle of friends and acquaintances too limited	0.20	0.03	0.33	0.04	0.22	0.03	0.37	0.03	0.21	0.02	0.35	0.03	0.40
I miss having people around me	0.14	0.03	0.30	0.04	0.22	0.03	0.46	0.03	0.18	0.02	0.38	0.03	0.52
Not living with partner, no daily contact with others	0.28	0.09	0.51	0.11	0.28	0.06	0.58	0.07	0.28	0.05	0.55	0.06	0.55
Not living with partner, daily contact with others	0.31	0.10	0.56	0.10	0.40	0.06	0.62	0.06	0.35	0.06	0.59	0.06	0.58
Living with partner	0.06	0.02	0.17	0.04	0.11	0.02	0.33	0.04	0.08	0.02	0.24	0.03	0.64
I often feel rejected	0.04	0.02	0.15	0.03	0.06	0.01	0.09	0.02	0.05	0.01	0.12	0.02	0.84
Social loneliness													
There is always someone I can talk to about my day-to-day problems †	0.07	0.02	0.23	0.04	0.15	0.02	0.17	0.02	0.10	0.02	0.20	0.02	0.77
There are plenty of people I can lean on when I have problems †	0.11	0.03	0.23	0.04	0.12	0.02	0.14	0.02	0.12	0.02	0.18	0.02	0.46
There are many people I can trust completely †	0.28	0.04	0.41	0.04	0.28	0.03	0.32	0.03	0.28	0.02	0.37	0.03	0.32
There are enough people I feel close to †	0.15	0.03	0.25	0.04	0.19	0.02	0.21	0.02	0.17	0.02	0.23	0.02	0.38
I can call on my friends whenever I need them †	0.14	0.03	0.36	0.04	0.18	0.02	0.22	0.03	0.16	0.02	0.28	0.02	0.70
Existential loneliness													
I am happy with the way I have lived my life †	0.13	0.03	0.15	0.03	0.14	0.02	0.16	0.02	0.14	0.02	0.16	0.02	0.11
There is a purpose to my life †	0.44	0.05	0.51	0.05	0.41	0.03	0.38	0.03	0.43	0.03	0.44	0.03	0.15
I feel helpless	0.04	0.02	0.05	0.02	0.04	0.01	0.08	0.02	0.04	0.01	0.06	0.01	0.09
I feel at the mercy of the world	0.29	0.04	0.33	0.04	0.45	0.03	0.33	0.03	0.36	0.03	0.33	0.03	0.13
I feel dead									0.01	0.01	0.02	0.01	0.29
The universe is full of meaning †	0.26	0.04	0.39	0.04	0.28	0.03	0.29	0.03	0.27	0.02	0.34	0.03	0.35
I feel that there is little point to life	0.10	0.03	0.10	0.03	0.11	0.02	0.11	0.02	0.11	0.02	0.11	0.02	0.00

Notes. N = 404. Written or digital questionnaire, n = 132; home or telephone interview, n = 272. Not living with partner, no daily contact with others, n = 86; not living with partner, daily contact with others, n = 87; living with partner, n = 231. Scores are reversed for positively phrased items marked with †. Items are dichotomous. For the item "I feel dead," the convergence criteria for the interaction model were not met. Effect size d is computed as the unstandardized regression coefficient (presented in Table S3) divided by 1.81 (Chinn, 2000). Cohen’s (1988) d = 0.2 is a small effect, 0.5 is a medium effect and 0.8 is a large effect.

Table S5. Correlations between variables included in the regression analysis (N = 404; after imputation of missing values)

	Emotional T1	Social T1	Existential T1	Emotional T0	Social T0	Existential T0	Living with partner T0	Network size T0	Daily contacted network member T0	Social participation frequency T0	Church attendance frequency T0	Mastery T0	Self-esteem T0	Number of chronic diseases T0	Physical functioning T0	Level of urbanity T0	Education level (years)	Income T0	Age T0	Female	Interval T0-T1 (years)	Variation interview date T1 (days)	Oral interview T1
Emotional T1	1																						
Social T1	0.747	1																					
Existential T1	0.846	0.837	1																				
Emotional T0	0.728	0.583	0.629	1																			
Social T0	0.538	0.620	0.548	0.726	1																		
Existential T0	0.629	0.615	0.670	0.838	0.832	1																	
Living with partner T0	-0.415	-0.254	-0.325	-0.423	-0.241	-0.341	1																
Network size T0	-0.080	-0.217	-0.161	-0.141	-0.250	-0.229	0.096	1															
Daily contacted network member T0	0.233	0.116	0.148	0.244	0.064	0.138	-0.608	-0.100	1														
Social participation frequency T0	-0.109	-0.159	-0.152	-0.118	-0.134	-0.190	0.083	0.219	-0.092	1													
Church attendance frequency T0	0.077	-0.048	-0.030	0.007	-0.027	-0.080	0.007	0.113	-0.009	0.365	1												
Mastery T0	-0.345	-0.320	-0.364	-0.307	-0.274	-0.377	0.120	0.088	-0.047	-0.049	-0.138	1											
Self-esteem T0	-0.306	-0.303	-0.343	-0.342	-0.325	-0.411	0.136	0.047	0.003	0.013	-0.094	0.497	1										
Number of chronic diseases T0	0.189	0.182	0.214	0.177	0.132	0.213	-0.082	-0.143	0.067	-0.106	0.025	-0.228	-0.204	1									
Physical functioning T0	-0.307	-0.272	-0.322	-0.268	-0.239	-0.320	0.156	0.078	-0.093	0.154	0.058	0.313	0.330	-0.382	1								
Level of urbanity T0	0.002	-0.045	0.018	0.032	0.040	0.062	-0.088	-0.085	0.024	-0.138	-0.189	0.070	0.051	-0.015	0.007	1							
Education level (years)	-0.122	-0.058	-0.112	-0.121	-0.104	-0.123	0.102	0.208	-0.136	0.089	-0.134	0.187	0.084	-0.161	0.195	0.081	1						
Income T0	0.022	0.035	-0.008	0.060	0.016	0.043	-0.213	0.053	0.044	0.085	-0.070	0.073	0.038	-0.105	0.097	0.133	0.346	1					
Age T0	0.227	0.154	0.225	0.246	0.219	0.265	-0.260	-0.071	0.166	-0.120	0.056	-0.239	-0.231	0.167	-0.414	0.010	-0.078	-0.023	1				
Female	0.151	0.059	0.164	0.189	0.096	0.199	-0.308	0.078	0.113	0.004	0.064	-0.210	-0.243	0.052	-0.148	0.050	-0.277	-0.124	0.095	1			
Interval T0-T1 (years)	-0.107	-0.065	-0.108	-0.130	-0.072	-0.080	0.012	-0.089	0.045	-0.027	-0.004	0.035	0.068	0.003	0.137	-0.145	-0.074	-0.022	-0.253	-0.044	1		
Variation interview date T1 (days)	0.066	0.028	0.073	0.056	0.029	0.042	0.016	0.140	-0.027	0.065	0.047	-0.061	-0.132	0.024	-0.046	-0.003	0.001	-0.033	0.100	0.087	-0.444	1	
Oral interview T1	0.091	-0.081	-0.003	0.134	0.097	0.111	-0.102	-0.023	0.046	0.025	0.078	-0.088	-0.078	0.000	-0.060	0.105	-0.065	-0.017	0.057	0.112	0.038	-0.151	1

References

Chinn, S. (2000). A simple method for converting an odds ratio to effect size for use in meta-analysis. *Statistics in Medicine, 19*(22), 3127-3131. doi:10.1002/1097-0258(20001130)19:22<3127::AID-SIM784>3.0.CO;2-M

Cohen, J. (1988). *Statistical power analysis for the behavioral sciences (second edition)*. Mahwah, NJ: Lawrence Erlbaum.
